# Quercetin ameliorates epithelial-mesenchymal transition and inflammation by targeting FSTL1 and modulating the NF-κB pathway in pulmonary fibrosis

**DOI:** 10.3389/fphar.2025.1594757

**Published:** 2025-07-30

**Authors:** Yuejiao Lan, Cuiting Dong, Mingda Wu, Ruichen Yuan, Kunpeng Yang, Zhen Yang, Yang Chen, Jingbin Zhang, Bingxue Qi, Xiaodan Lu

**Affiliations:** ^1^ Changchun University of Chinese Medicine, Changchun, Jilin, China; ^2^ Jilin Province People’s Hospital, Changchun, Jilin, China

**Keywords:** pulmonary fibrosis, quercetin, FSTL1, NF-κB, EMT, inflammation

## Abstract

**Background:**

Idiopathic pulmonary fibrosis (IPF) is a progressive disorder characterized by chronic inflammation and pathological lung remodeling driven by excessive extracellular matrix deposition. While the flavonol quercetin exhibits established anti-inflammatory and antioxidant properties, its therapeutic mechanisms against IPF—particularly regarding epithelial-mesenchymal transition (EMT) and inflammation regulation via the follistatin-like 1 (FSTL1)/nuclear factor kappa B (NF-κB) axis—remain incompletely elucidated. This study therefore investigates quercetin’s capacity to mitigate pulmonary fibrosis through targeted modulation of the FSTL1/NF-κB pathway.

**Methods:**

A bleomycin (BLM)-induced pulmonary fibrosis mouse model and Transforming Growth Factor Beta 1 (TGF-β1)-induced EMT models in A549 and BEAS-2B cells were employed. The therapeutic effects of quercetin were assessed through H&E, Masson, Sirius red staining, immunofluorescence, quantitative real-time PCR (qRT-PCR), and Western blotting. The role of FSTL1 and NF-κB signaling in the anti-fibrotic effects of quercetin was evaluated using FSTL1 knockdown.

**Results:**

*In vivo* studies have shown that BLM-induced pulmonary fibrosis and inflammation significantly increased the deposition of extracellular matrix and the levels of interleukin-1 beta (IL-1β), monocyte chemoattractant protein-1 (MCP-1), and interleukin 6 (IL-6), all of which were markedly reduced by quercetin administration. *In vitro* experiments revealed that quercetin suppressed TGF-β1-induced EMT and inflammation. Importantly, FSTL1 knockdown diminished the anti-inflammatory and anti-EMT effects of quercetin.

**Conclusion:**

Quercetin exerts its protective effects against pulmonary fibrosis by suppressing FSTL1 expression and modulating the NF-κB signaling pathway, thereby inhibiting both inflammation and EMT process.

## Graphical Abstract

**Figure:**
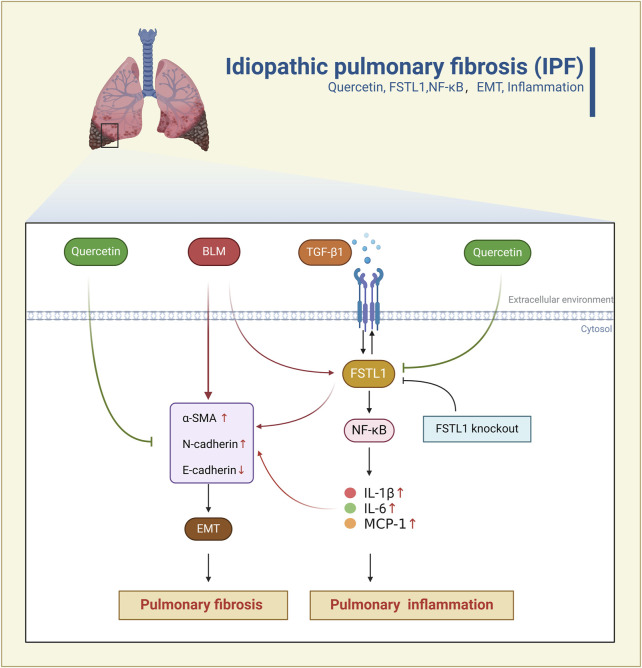
Schematic representation of Quercetin’s anti-fibrotic effects through the FSTL1/NF-κB pathway. BLM/TGF-β1 induces epithelial-mesenchymal transition (EMT), inflammation, and pulmonary fibrosis by activating the FSTL1/NF-κB signaling pathway. Quercetin inhibits the FSTL1/NF-κB pathway, thereby alleviating EMT, inflammation, and suppressing pulmonary fibrosis. FSTL1 knockout abolishes the anti-fibrotic and anti-inflammatory effects of Quercetin.

## 1 Introduction

Idiopathic pulmonary fibrosis (IPF) is a progressive interstitial lung disease, characterized by excessive accumulation of extracellular matrix (ECM), which leads to irreversible lung damage and impaired pulmonary function. The prevalence of IPF is estimated to be 1.6-1.7 cases per 1,000 individuals, with a median survival rate of 3–5 years following diagnosis (Sgalla et al., 2018; Maher et al., 2021). Currently, the mainstay of treatment includes antifibrotic drugs such as Pirfenidone and Nintedanib, which have been shown to slow the decline in lung function and reduce the risk of acute exacerbations. However, these medications can cause serious adverse side effects including gastrointestinal issues like diarrhea for Nintedanib, and for Pirfenidone, side effects may include photosensitivity, nausea, and liver function abnormalities (Gu et al., 2021; Man et al., 2024). Therefore, an in-depth investigation into the pathogenesis of IPF, alongside the development of novel therapeutic agents, is urgently required to improve clinical outcomes. Aberrant epithelial-mesenchymal transition (EMT) plays a pivotal role in the pathogenesis of pulmonary fibrosis. This process involves the reorganization of the cytoskeleton in epithelial cells and the disruption of junctional complexes, leading to morphological alterations and enhanced cellular motility (Yao et al., 2024). Studies have demonstrated that cytokines, including transforming growth factor-β (TGF-β), tumor necrosis factor (TNF-α), and interleukin-1β (IL-1β), can induce EMT in mesenchymal cells. Furthermore, nuclear Factor kappa-B (NF-κB) is activated in response to inflammatory signals, which leads to the expression of Snail, a transcription factor that promotes EMT (Liu et al., 2021). Moreover, transforming growth factor-β1 (TGF-β1) is a pivotal cytokine that, in conjunction with NF-κB activation, enhances EMT and fibrosis (Alyaseer et al., 2020; Sisto et al., 2021). In addition, Follistatin-like 1 (FSTL1) is a secreted glycoprotein that plays a crucial role in various biological processes, including inflammation, tissue repair, and fibrosis. It is involved in the regulation of cell proliferation, differentiation, and apoptosis. In the context of IPF, FSTL1 is induced by TGF-β1 (Zhang et al., 2022) and is upregulated in IPF lung tissues. This upregulation promotes the activation of pulmonary fibroblasts and the production of ECM components, which are key factors in the progression of fibrosis (Sun et al., 2023). The potential of FSTL1 as a therapeutic target is underscored by the effectiveness of neutralizing antibodies in reducing fibrosis in experimental models (Jin et al., 2020). Consequently, this study aims to explore the role of FSTL1 in the EMT process of pulmonary fibrosis and its regulatory mechanisms on the NF-κB pathway, providing a novel perspective on the development of therapeutic strategies for IPF.

Quercetin, a flavonoid found in many fruits and vegetables, exhibits therapeutic potential in diseases through its antioxidant, anti-inflammatory, and anticancer properties. It has been shown to have antioxidant and anti-inflammatory effects in conditions like polycystic ovary syndrome and various cancers (Jian et al., 2024), as well as neuroprotective effects in Parkinson’s disease. Moreover, in fibrotic diseases, quercetin mitigates hepatic fibrosis by modulating glycolysis in hepatic stellate cells, reducing neutrophil infiltration, and improving liver function markers (Wang et al., 2024). In addition, quercetin reduces cellular senescence in alveolar epithelial cells and macrophages, inhibits the senescence-associated secretory phenotype (SASP) (Geng et al., 2022) and reduces the protein expression of NF-κB signaling pathway in lung tissue (Yousefi Zardak et al., 2024). Since NF-κB plays a key role in regulating the expression of FSTL1(Mattiotti et al., 2018; Hu et al., 2019; Yan et al., 2024), quercetin may downregulate FSTL1 expression to inhibit pulmonary fibrosis by inhibiting the NF-κB signaling pathway. Therefore, this study aims to observe the effects of quercetin on pulmonary fibrosis in BLM-induced pulmonary fibrosis mouse model and TGF-β1-induced pulmonary fibrosis in A549 and BEAS-2B cells, as well as its impact on EMT and inflammation during the fibrosis process.

## 2 Materials and methods

### 2.1 Establishment of the pulmonary fibrosis model and animal grouping

Forty 6-8-week-old male C57BL/6J mice, weighing approximately 20–30 g, were housed at the Center for Transgenic Animal Experimentation, Northeast Normal University (Changchun, Jilin, China). The mice were kept under specific pathogen-free (SPF) conditions at a controlled temperature (25°C ± 2°C), with a 12-h light/dark cycle, and had *ad libitum* access to water and a standard diet. The animals were randomly divided into four groups: control, BLM, BLM with low-dose quercetin (QR, 50 mg/kg), and BLM with high-dose QR (100 mg/kg), with 10 mice per group. The dose selection for quercetin was informed by established literature (Zhang et al., 2018; Wu et al., 2024), wherein we designated 100 mg/kg as the primary treatment group and included 50 mg/kg as the lower-dose cohort (slightly below the fully effective dose) to monitor dose-response trends. Pulmonary fibrosis was induced by administering BLM (5.0 mg/kg, HY-17565, MedChem Express, Princeton, NJ, United States) via intratracheal instillation under isoflurane anesthesia. The treatment groups received QR (HY-18085, MedChem Express, Princeton, NJ, United States) by daily oral gavage for 21 days. All experiments were conducted in accordance with ethical guidelines, and the procedures were approved by the Laboratory Animal Ethics Committee of Changchun University of Traditional Chinese Medicine (approval number: 2024580).

### 2.2 H&E staining

Mouse lung tissues were taken, rinsed in saline and fixed in 4% paraformaldehyde fixation. After gradient ethanol dehydration and xylene transparency, the tissue was immersed in paraffin embedding and 4 μm sections were made. The slides were dewaxed and hydrated, stained with hematoxylin and then restained with eosin (H&E, C0105s, Beyotime Biotechnology, Shanghai, China), dehydrated and transparent, and finally the slides were fixed with neutral adhesive and analyzed under a light microscope (NIB610, Nexcope, Ningbo, China). The extent of pulmonary fibrosis in each image was quantified as a numerical form of inflammation score and a numerical form of fibrosis score for each image, ranging from 0 (normal) to 3 (more severe), according to the criteria described by (Szapiel et al., 1979). Level 0, normal tissues without alveolitis or fibrosis; level 1, mild alveolitis or fibrosis with <20% lesions of the lung; level 2, moderate alveolitis or fibrosis with 20%–50% lesions of the lung; level 3, severe alveolitis or fibrosis with >50% lesions of the lung.

### 2.3 Masson’s trichrome staining

Sections (approximately 4 μm) were stained with a Masson staining kit to detect collagen fibres (C0189, Beyotime, Shanghai, China). The dried sections were deparaffinized by immersion in xylene and rehydrated. Then the nuclei were stained with Weigert’s iron hematoxylin for 5 min, and then the nuclei were differentiated with 1% hydrochloric acid in alcohol and rinsed under running water to return the blue color. After that, the sections were stained with reichhorn red acidic re-staining solution for 10 min, differentiated with 1% phosphomolybdic acid aqueous solution for 3 min, then re-stained by aniline blue for 5 min, and finally rinsed with 0.2% glacial acetic acid aqueous solution. After dehydration, transparency and sealing, the sections were examined under a light microscope (NIB610, Nexcope, Ningbo, China) and photographed using Image-Pro Plus 6.0.

### 2.4 Sirius red staining

Initially, tissue sections are deparaffinized and rehydrated. They are then incubated in a 0.1% Sirius Red solution (365548, Sigma, Darmstadt, Germany) that is dissolved in saturated aqueous picric acid (P6744,Sigma, Darmstadt, Germany) for approximately 1 h. After incubation, the sections are washed in acidified water containing 0.5% hydrogen chloride to remove excess dye. Subsequently, the sections were dehydrated through a graded series of ethanol and cleared with xylene before being mounted with a cover slip. Finally, the results were observed under a light microscope (NIB610, Nexcope, Ningbo, China) and photographed in order to record the results using Image-Pro Plus 6.0.

### 2.5 Cell culture and transfection

Human lung epithelial A549 (CL-0016, Procell, Wuhan, China) and BEAS-2B (CL-0496, Procell, Wuhan, China) cell lines were purchased from Pronosay. All cell lines included in this study were characterized by STR profiling and tested for *mycoplasma* contamination. Cells were cultured in DMEM medium (PM150210, Vazyme, Nanjing, China) containing 10% fetal bovine serum (164210-50, Vazyme, Nanjing, China), penicillin/streptomycin, and routinely cultured at 5% CO2, 37°C. The cells were regular in morphology without aggregation. When the cell fusion reached 70%∼80%, the cells were digested and passaged.

FSTL1 knockdown sgRNA1 sequence (CAT​CTC​GAT​GCA​GTT​CAC​AG), sgRNA2 sequence (gct​cgc​gct​cgc​gct​GGT​GG) were cloned into the knockdown px330-CRISPR-Cas9 vector, respectively, and the constructed px330-CRISPR-Cas9 expression vector was transformed and extracted into plasmids to obtain high quality plasmids. Lipofectamine 3000 (L3000015, Thermo Fisher Scientific, Waltham, MA, United States) was used to transfect the plasmid into A549 cells. The construct targeting FSTL1 overexpression (NM_007085.5 CD region sequence) was cloned into the pLV3-CMV-FSTL1(human)-tdTomato-Puro expression vector, and the construct was plasmid transformed and extracted to obtain high-quality plasmid DNA. The plasmid was transfected into A549 cells using the Lipofectamine 3000 to transfect the plasmid into A549 cells.

### 2.6 Immunofluorescence assay

A549 or BEAS-2B cells were inoculated in six-well plates and fixed in 4% paraformaldehyde for 20 min. Paraffin sections were then subjected to dewaxing and antigen repair treatment, followed by infiltration of 0.5% Triton X-100 (9002-93-1, Coolaber, Beijing, China) for 5 min and then closed with 2% bovine serum albumin (BSA) (A8020, Solarbio, Beijing, China) for 45 min. The cells were then incubated with FSTL1 (1:1600 dilution, 20182-1-AP, Proteintech, Wuhan, China) and α-SMA primary antibody (1:400 dilution, 67735-1-Ig, Proteintech, Wuhan, China) at 4°C overnight. After washing the cells or sections with PBS for 5 min, which was performed three times to remove unbound antibodies, the cells were incubated with CoraLite^®^ Plus 488-labeled goat anti-rabbit secondary antibody (RGAR002, Proteintech, Wuhan, China) and CoraLite^®^ Plus 594-labeled goat anti-mouse secondary antibody (RGAM004, Proteintech, Wuhan, China) were incubated at room temperature for 1 h. The nuclei were finally stained with DAPI dye (C1005, Beyotime Biotechnology, Shanghai, China) and finally stained with anti-fade sealer. Images were captured using a light microscope (1X71, OLYMPUS, Tokyo, Japan).

### 2.7 Reverse transcription quantitative real-time polymerase chain reaction (RT-qPCR)

Total RNA was isolated from lung tissues and A549 and BEAS-2B cells using Trizol reagent (R401-01, Vazyme, Nanjing, China), and a total of 1 μg of isolated RNA was subjected to reverse transcription experiments using the Reverse Transcription Kit (R323-01, Vazyme, Nanjing, China), and the experimental system was 37°C for 15 min followed by 85°C for 5 s. The qPCR assay was performed using SYBR Green Real-Time Fluorescence PCR Kit (Q711-02, Vazyme, Nanjing, China) on a 7500 Fast Real-Time PCR System (Gentier 96R, NANBEI Co., Ltd., Zhengzhou, China). Relative gene expression was calculated using the 2^−ΔΔCT^ method, normalizing to the housekeeping gene 18s. Each sample was analyzed in triplicate to ensure accuracy. The primer sequences are as Table 1.

**TABLE 1:** Sequences of the primers for qRT-PCR.

Primer name	Forward primer (5′-3′)	Reverse primer (5′-3′)	Species
α-SMA	TGC​CAA​CAA​CGT​CAT​GTC​G	CAG​CGC​GGT​GAT​CTC​TTT​CT	Human
FSTL1	CCC​AGT​TGT​TTG​CTA​TCA​GTC​C	TGT​AGT​TGC​TGC​CTT​TAG​AGA​AC	Human
E-Cadherin	CGA​GAG​CTA​CAC​GTT​CAC​GG	GGG​TGT​CGA​GGG​AAA​AAT​AGG	Human
N-Cadherin	AGC​CAA​CCT​TAA​CTG​AGG​AGT	GGC​AAG​TTG​ATT​GGA​GGG​ATG	Human
IL-6	CCT​GAA​CCT​TCC​AAA​GAT​GGC	TTC​ACC​AGG​CAA​GTC​TCC​TCA	Human
MCP-1	CAG​CCA​GAT​GCA​ATC​AAT​GCC	TGG​AAT​CCT​GAA​CCC​ACT​TCT	Human
IL-1β	ATG​ATG​GCT​TAT​TAC​AGT​GGC​AA	GTC​GGA​GAT​TCG​TAG​CTG​GA	Human
18s	CAT​TCG​AAC​GTC​TGC​CCT​AT	GAT​GTG​GTA​GCC​GTT​TCT​CA	Human
α-SMA	CTA​CGA​ACT​GCC​TGA​CGG​G	GCT​GTT​ATA​GGT​GGT​TTC​GTG​G	Mouse
FSTL1	CAC​GGC​GAG​GAG​GAA​CCT​A	TCT​TGC​CAT​TAC​TGC​CAC​ACA	Mouse
E-Cadherin	CAG​CCT​TCT​TTT​CGG​AAG​ACT	GGT​AGA​CAG​CTC​CCT​ATG​ACT​G	Mouse
N-Cadherin	CTC​CAA​CGG​GCA​TCT​TCA​TTA​T	CAA​GTG​AAA​CCG​GGC​TAT​CAG	Mouse
IL-6	ATC​CAG​TTG​CCT​TCT​TGG​GAC​TGA	TAA​GCC​TCC​GAC​TTG​TGA​AGT​GGT	Mouse
MCP-1	TAA​AAA​CCT​GGA​TCG​GAA​CCA​AA	GCA​TTA​GCT​TCA​GAT​TTA​CGG​GT	Mouse
IL-1β	GAA​ATG​CCA​CCT​TTT​GAC​AGT​G	TGG​ATG​CTC​TCA​TCA​GGA​CAG	Mouse
COL1	CCA​AGA​AGA​CAT​CCC​TGA​AGT​CA	TGCACGTCATCGCACACA	Mouse
18s	CGC​CGC​TAG​AGG​TGA​AAT​TC	CGA​ACC​TCC​GAC​TTT​CGT​TCT	Mouse

### 2.8 Western blotting

Total proteins from lung tissues and cells were extracted using RIPA lysis buffer containing protease inhibitors (PR20016, Proteintech, Wuhan, China), and proteins were quantified using a bicinchoninic acid (BCA) kit (PK10026, Proteintech, Wuhan, China). Equal amounts of protein were then separated on 10% SDS-PAGE (P1015, Solarbio, Beijing, China). After electrophoresis, the proteins were transferred onto a PVDF membrane (A30788001, Cytiva, MA, United States) and placed in 5% skimmed milk (BS102, Biosharp, LJK Technology Co., Ltd., Hefei, China) powder at room temperature for 1-2 h. The membrane was then incubated with anti-FSTL1 (20182-1-AP, Proteintech, Wuhan, China), alpha-smooth muscle actin (α-SMA,14395-1-AP, Proteintech, Wuhan, China), E-Cadherin (20874-1-AP, Proteintech, Wuhan, China), N-Cadherin (13116, Cell Signaling Technology (CST), Massachusetts, United States), NF-κB (T55034, Abmark, Shanghai, China), phosphorylated nuclear factor kappa-B (pNF-κB, 3033, Cell Signaling Technology, Danvers, MA, United States), glyceraldehyde-3-phosphate dehydrogenase (GAPDH, HRP-60004, Proteintech, Wuhan, China), primary antibodies were incubated overnight at 4°C, and GAPDH was used as a control. On day 2, the membrane was washed three times by TBST for 5 min/time, and the corresponding HRP-coupled secondary antibodies (RGAM004, RGAR002, Proteintech, Wuhan, China) were added and incubated at room temperature for 2 h, and the membrane was washed by TBST shaking bed oscillation for 10 min × 3 times. The protein bands were visualized using a Fully automated chemiluminescence gel imaging system (O1600MF, Guangzhou GuangYi Biohnology Co., Ltd., Guangzhou, Guangdong), and then the intensity of the bands was quantified using ImageJ software.

### 2.9 Bioinformation analysis

The current exploration of FSTL1 expression was performed using the dataset GSE70866 from the Gene Expression Omnibus (GEO) database, including 20 normal samples (GSM1820719 to GSM1820738) and 112 samples from patients with idiopathic pulmonary fibrosis (IPF), was used in this study. Data analysis was performed using R software (version 4.4.0) loaded with the packages including dplyr, ggplot2 and ggpubr. The expression data of FSTL1 gene were firstly screened from the dataset, and the samples were divided into normal and IPF patient groups, and the respective expression values were extracted and combined into one data frame. The Wilcoxon rank sum test was used to statistically compare the two groups, and box-and-line plots were used to visualize the expression differences, and statistical significance labels were added. To further evaluate the association between FSTL1 and NF-κB pathway genes, Pearson correlation analysis (using the Hmisc package) was employed, generating a correlation coefficient matrix and heatmap (using the pheatmap package) with a significance threshold set at *P < 0.05.*

### 2.10 Statistical analysis

All data are expressed as mean ± SEM and were analyzed using Prism GraphPad Software version 9.0. Data between two groups were analyzed by Student’s t-test, while one-way analysis of variance (ANOVA) was used to test the analysis of differences between multiple groups. *P < 0.05* was considered statistically significant.

## 3 Results

### 3.1 Quercetin ameliorated lung damages and inflammation in BLM-induced pulmonary fibrosis *in vivo*

**FIGURE 1:**
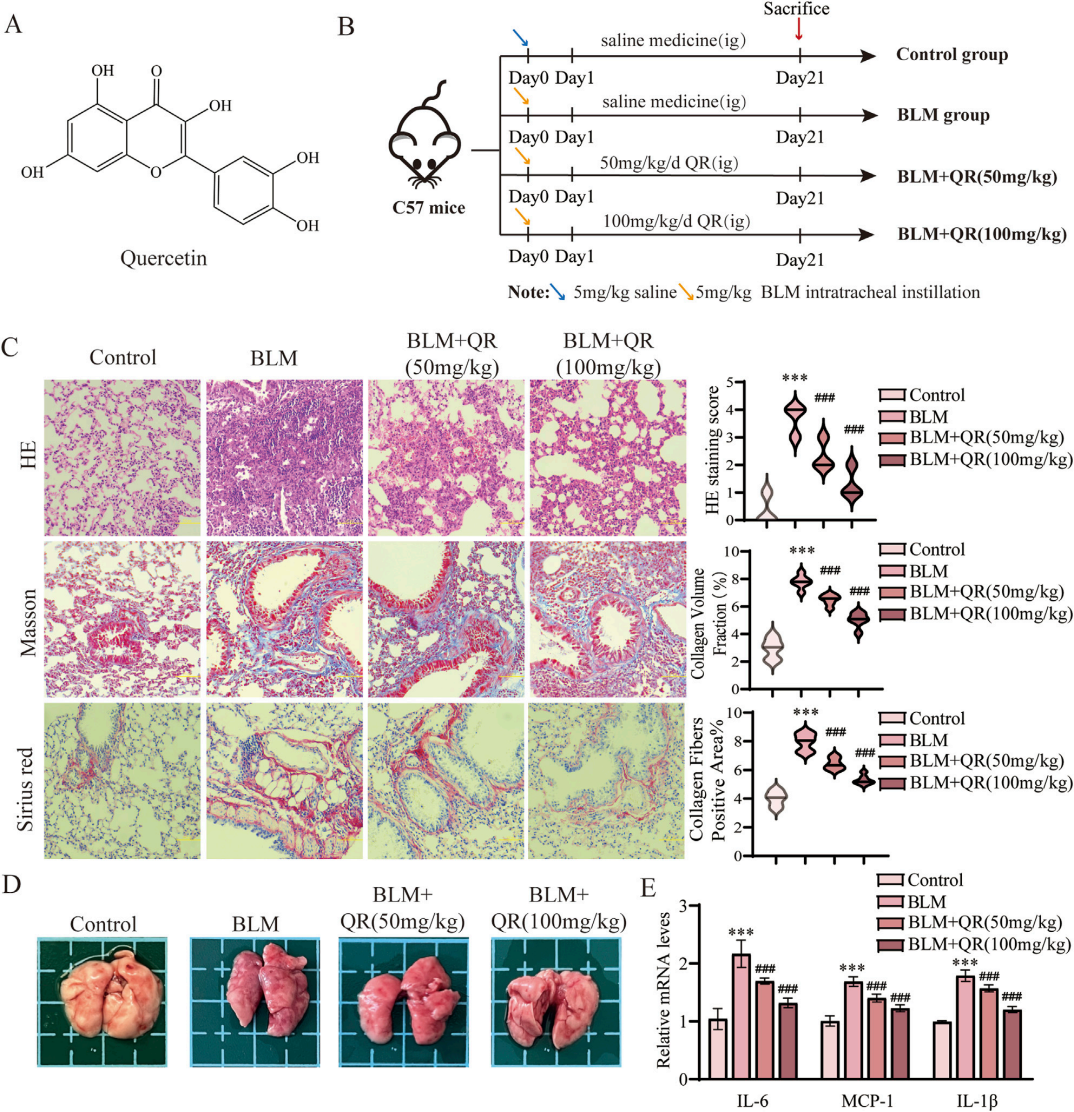
Quercetin alleviates BLM-induced pulmonary fibrosis and inflammation in pulmonary fibrosis mouse model. **(A)** The molecular formula of quercetin. **(B)** Schematic representation of the animal experimental model. **(C)** Representative images of H&E, Masson, and Sirius Red staining in lung tissue from four groups, along with quantitative analysis of H&E, Masson, and Sirius Red staining (n = 6). **(D)** Lung image plots. **(E)** Real-time qPCR analysis of mRNA expression, relative levels of IL-6, IL-1β, and MCP-1 (n = 3). Data are presented as means ± SEM; *P < 0.05, **P < 0.01, and ***P < 0.001 compared to the control group; ^*#*^ *P < 0.05,* ^*##*^ *P < 0.01*, and ^*###*^ *P < 0.001* compared with the BLM group. Abbreviations: BLM, Bleomycin; H&E, Hematoxylin and Eosin; IL-6, Interleukin-6; IL-1β, Interleukin-1β; QR, Quercetin. Ig, Intragastric Administration; MCP-1, Monocyte Chemoattractant Protein-1.

The chemical structure of quercetin is depicted in Figure 1A. It consists of two benzene rings connected via an oxygen-containing pyran ring. The molecular formula of quercetin is C_15_H_10_O_7_, and it contains five hydroxyl groups. These hydroxyl groups contribute to its potent antioxidant activity, which is estimated to be 50 times more potent than vitamin E and 20 times more potent than vitamin C (Michala and Pritsa, 2022). This antioxidant capacity enables quercetin to directly scavenge free radicals, support enzymatic elimination, and inhibit radical formation. To evaluate quercetin’s regulatory effects on BLM-induced pulmonary fibrosis and inflammation, quercetin was administered intragastrically to C57BL/6 mice following intratracheal BLM instillation. Treatment was administered at high (100 mg/kg) and low (50 mg/kg) doses to assess its therapeutic impact on pulmonary fibrosis. Mice were euthanized 21 days post-treatment (Figure 1B). Comparison to the control group, BLM-induced fibrosis in mice resulted in substantial widening of alveolar septa, thickening of alveolar walls, accumulation of fibrotic exudates in alveolar spaces, and increased infiltration of mononuclear cells throughout the lung parenchyma. Quercetin treatment significantly alleviated these pathological changes (Figure 1C). Masson’s trichrome staining highlighted marked fibrotic changes in the model group, with notable collagen fiber deposition that appeared blue under staining, indicating severe fibrosis (Figure 1C). Sirius Red staining results reveal that the control group shows minimal collagen deposition, as indicated by the faint red coloration in the lung tissue sections. In contrast, the BLM group exhibited a marked increase in red staining, which highlights the extensive accumulation of collagen. The group treated with low-concentration quercetin demonstrated a moderate reduction in staining intensity, suggesting partial attenuation of fibrotic changes. Finally, the high-concentration quercetin group exhibits the most pronounced decrease in red coloration, indicating a substantial reduction in collagen deposition and a potential therapeutic effect against BLM-induced pulmonary fibrosis (Figure 1C). Quercetin treatment reversed collagen deposition in a dose-dependent manner, demonstrating comparable regulatory effects on fibrotic markers to the positive control drug Pirfenidone (Tanner et al., 2023; Xie et al., 2023). Both agents significantly inhibited pulmonary fibrosis, highlighting quercetin’s potential as a therapeutic agent for this condition. Dispersed deep red hemorrhagic spots were observed in the lungs of BLM-treated mice (Figure 1D). RT-qPCR analysis of lung tissue revealed that BLM administration significantly upregulated pro-inflammatory markers MCP-1, IL-6, and IL-1β. Quercetin effectively reduced the levels of these inflammatory markers, as shown in Figure 1E.

### 3.2 Quercetin attenuated the BLM-induced EMT and by suppressing FSTL1 expression and modulating the NF-κB signaling pathway *in vivo*

**FIGURE 2:**
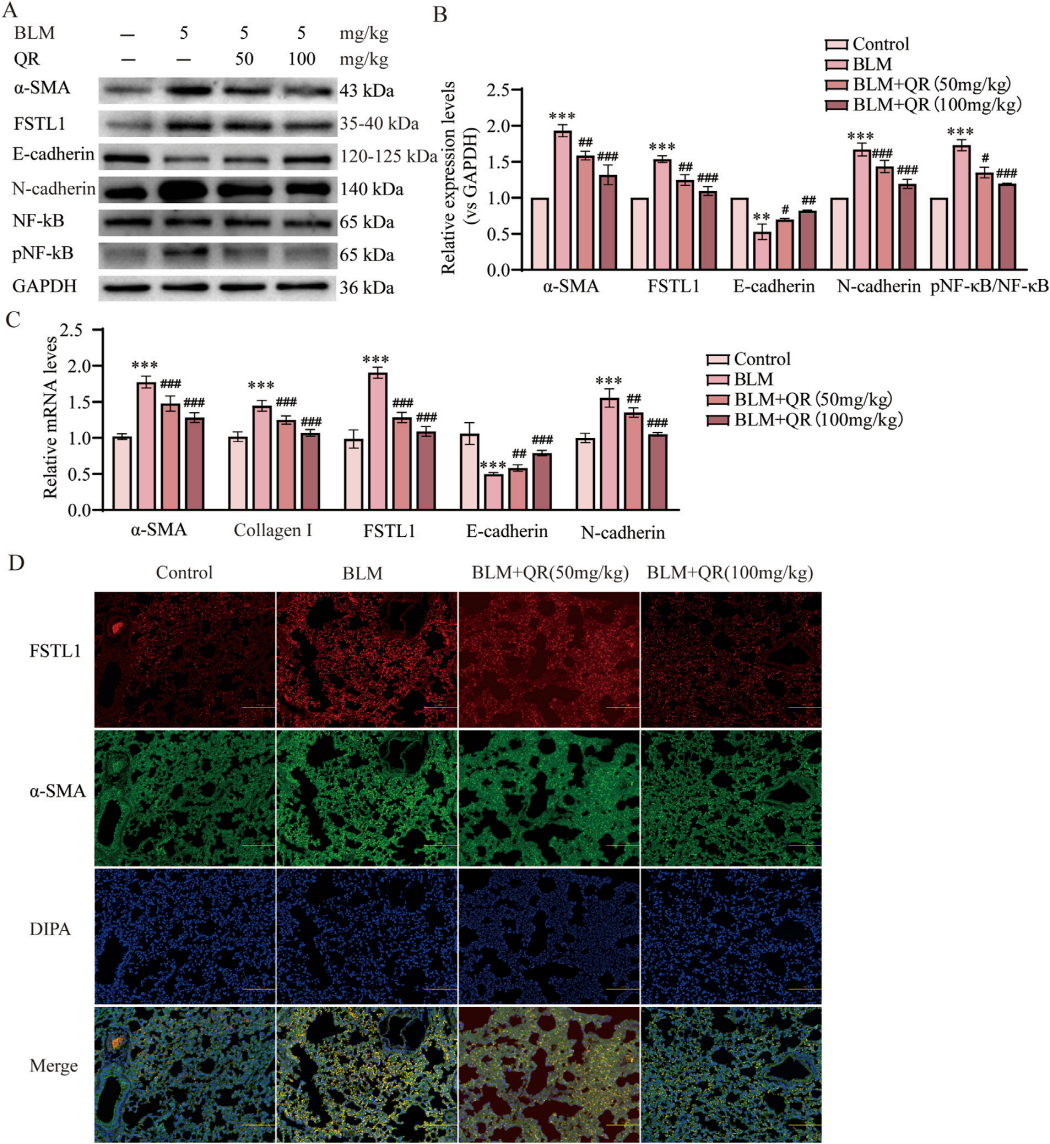
Quercetin alleviates BLM-induced EMT via FSTL1/NF-κB signaling pathway in a pulmonary fibrosis mouse model. **(A)** Protein expression levels of α-SMA, FSTL1, E-cadherin, N-cadherin, total NF-κB, and phosphorylated NF-κB were determined using Western blotting. **(B)** Quantitative analysis of α-SMA, FSTL1, E-cadherin, N-cadherin, total NF-κB, and phosphorylated NF-κB, with GAPDH serving as a loading control (n = 3). **(C)** Real-time qPCR analysis of mRNA expression, relative levels of α-SMA, Collagen I, FSTL1, E-cadherin, and N-cadherin (n = 3). **(D)** Double immunofluorescence staining for FSTL1 and α-SMA in mouse lungs, with a scale bar of 100 μm (n = 3). Data are presented as means ± SEM; **P < 0.01 and ***P < 0.001 compared to the control group; ^*#*^ *P < 0.05*, ^##^ *P < 0.01*, and ^###^ *P < 0.001* compared to the BLM group. Abbreviations: BLM, Bleomycin; FSTL1, Follistatin-like 1; NF-κB, Nuclear Factor kappa B; α-SMA, Alpha-Smooth Muscle Actin; TGF-β1, Transforming Growth Factor Beta 1; QR, Quercetin; pNF-κB, phosphorylated Nuclear Factor kappa-B; GAPDH, Glyceraldehyde-3-Phosphate Dehydrogenase.

It has been well-demonstrated that epithelial-mesenchymal transition (EMT) plays a critical role in the progression of PF. Alpha-Smooth Muscle Actin (α-SMA) serves as a mesenchymal marker, with its increased expression signifying the transition of epithelial cells to mesenchymal cells, a hallmark of EMT. The progression of EMT is characterized by the downregulation of the epithelial adhesion protein E-cadherin and the upregulation of the mesenchymal marker N-cadherin. In this study, Western blot analysis revealed a significant increase in the expression of α-SMA and N-cadherin, accompanied by a reduction in E-cadherin expression in the group treated with intratracheal BLM. In contrast, in the quercetin-treated group, the BLM-induced reduction in E-cadherin was prevented, and the increases in α-SMA and N-cadherin were attenuated (Figures 2A,B). Similarly, qPCR analysis of lung tissue demonstrated trends that were consistent with the Western blot results (Figure 2C). Further studies have indicated that FSTL1 activates the NF-κB signaling pathway, leading to increased expression of inflammatory and catabolic factors, such as IL-1β, TNF-α, and IL-6. In macrophages with FSTL1 deficiency, pro-inflammatory M1 polarization is suppressed, and activation of the NF-κB pathway is reduced (Rao et al., 2022). *In vivo* Western blot and qPCR results further demonstrated that FSTL1 expression and the pNF-κB/NF-κB ratio were elevated in the BLM group, suggesting that the FSTL1/NF-κB signaling pathway contributes to the progression of pulmonary fibrosis. Quercetin was found to partially reverse these changes (Figures 2A,B). Immunofluorescence analysis of lung tissue sections revealed a significant increase in FSTL1 (green fluorescence) and α-SMA (red fluorescence) in the BLM group. Under high magnification, FSTL1 fluorescence was uniformly distributed along the alveolar walls and stromal regions, while α-SMA fluorescence was predominantly localized to smooth muscle cells surrounding the alveoli and certain stromal cells. Immunofluorescence analysis revealed the treatment effects on fibrosis markers. The control group exhibited minimal red fluorescence for FSTL1 and green fluorescence for α-SMA. In contrast, the BLM-induced PF group showed a significant increase in both FSTL1 and α-SMA fluorescence, indicating enhanced expression of these proteins associated with fibrosis. The group treated with quercetin post-BLM exhibited a moderate reduction in FSTL1 and α-SMA fluorescence compared to the BLM group, suggesting a partial reversal of fibrotic changes (Figure 2D). Treatment with quercetin significantly reduced the fluorescence levels of both FSTL1 and α-SMA, suggesting that quercetin’s regulatory effects on pulmonary fibrosis may involve FSTL1.

### 3.3 The expression of FSTL1 was increased in TGF-β1-induced EMT *in vitro*

**FIGURE 3:**
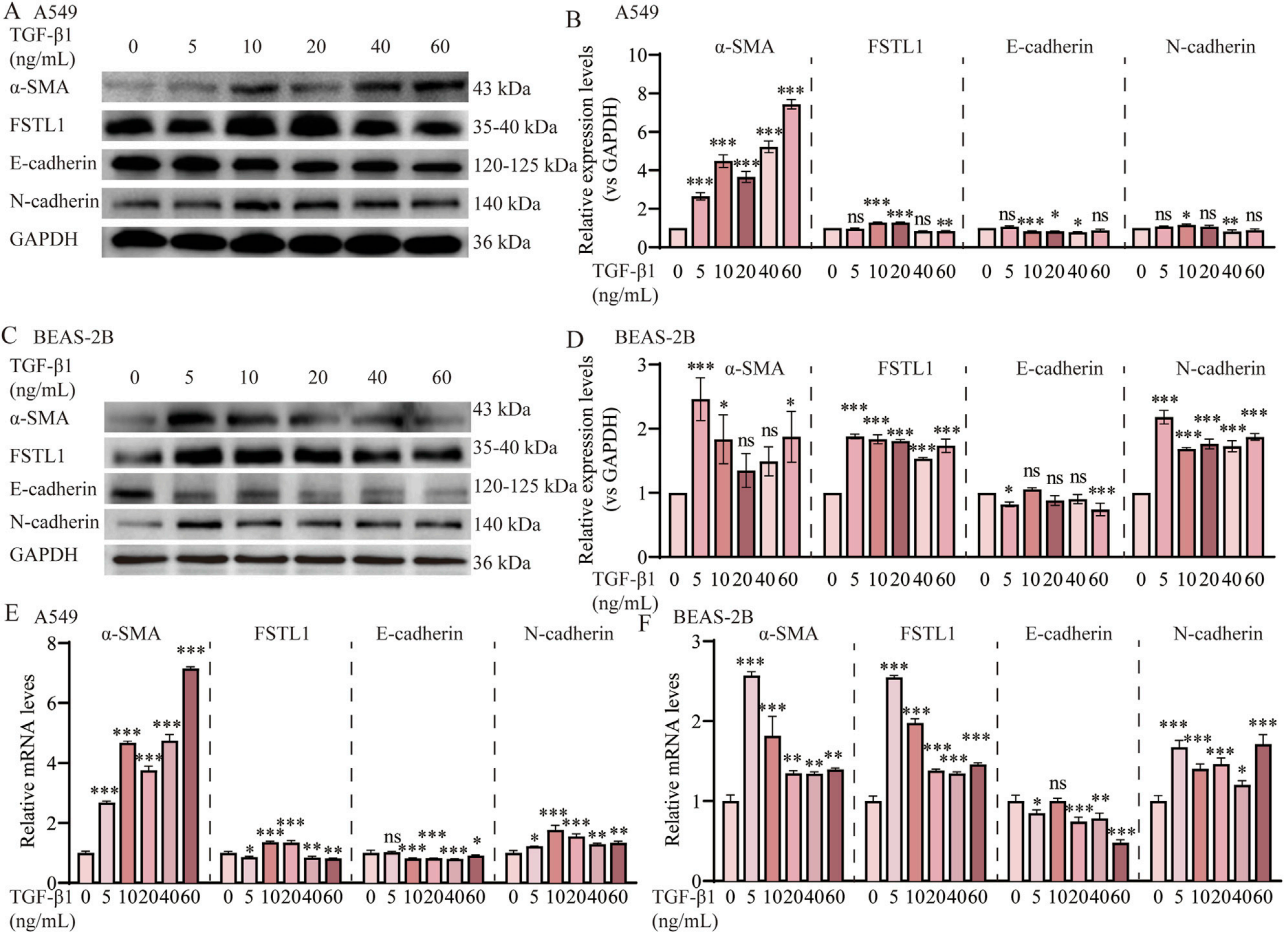
TGF-β1 induces EMT via the FSTL1/NF-κB signaling pathway *in vitro*. **(A)** Protein expression levels of α-SMA, FSTL1, E-cadherin, N-cadherin in A549 cells were assessed by Western blotting. **(B)** Quantitative analysis of α-SMA, FSTL1, E-cadherin, N-cadherin proteins was performed, with GAPDH used as a loading control in A549 cells (n = 3). **(C)** Protein expression levels of α-SMA, FSTL1, E-cadherin, N-cadherin in BEAS-2B cells were assessed by Western blotting. **(D)** Quantitative analysis of α-SMA, FSTL1, E-cadherin, N-cadherin proteins was performed, with GAPDH used as a loading control in BEAS-2B cells (n = 3). **(E)** Real-time qPCR analysis of mRNA expression, relative levels of α-SMA, FSTL1, E-cadherin, and N-cadherin in A549 cells (n = 3). **(F)** Real-time qPCR analysis of mRNA expression, relative levels of α-SMA, FSTL1, E-cadherin, and N-cadherin in BEAS-2B cells (n = 3). Data are presented as means ± SEM; *P < 0.05, **P < 0.01, and ***P < 0.001 compared to the TGF-β1 0 group. Abbreviations: TGF-β1, Transforming Growth Factor Beta 1; FSTL1, Follistatin-like 1; α-SMA, Alpha-Smooth Muscle Actin; GAPDH, Glyceraldehyde-3-Phosphate Dehydrogenase.

FSTL1, a glycoprotein known to be induced by TGF-β1, was assessed for its activation in A549 and BEAS-2B cells upon exposure to increasing concentrations of TGF-β1. Western blot analysis (Figures 3A,C) and subsequent quantification (Figures 3B,D) demonstrated a significant upregulation of FSTL1 in A549 cells at 10 ng/mL and 20 ng/mL TGF-β1, as well as in BEAS-2B cells at 5 ng/mL and 10 ng/mL TGF-β1. This suggests that TGF-β1 plays a regulatory role in FSTL1 expression, which may be integral to the pathogenesis of pulmonary fibrosis. In A549 cells, α-SMA expression escalated with increasing TGF-β1 concentrations, while E-cadherin levels exhibited a pronounced decrease at 10 ng/mL, stabilizing at higher concentrations. Consequently, 10 ng/mL TGF-β1 was determined to be optimal for establishing an EMT model in A549 cells. In contrast, BEAS-2B cells responded to 5 ng/mL TGF-β1 with a more than two-fold α-SMA expression increase relative to control levels, with a diminishing return observed at higher concentrations. E-cadherin expression was significantly downregulated at 5 ng/mL, as shown in Figure 3E,F. Therefore, 5 ng/mL TGF-β1 was chosen for the development of an *in vitro* pulmonary fibrosis model in BEAS-2B cells. The presented data underscore the differential response of FSTL1 and EMT-related markers to TGF-β1 in A549 and BEAS-2B cells, providing a basis for further investigation into the molecular mechanisms underlying TGF-β1-mediated fibrosis.

### 3.4 Overexpression of FSTL1 induced TGF-β1 expression and inflammation, and EMT in A549 cells

**FIGURE 4:**
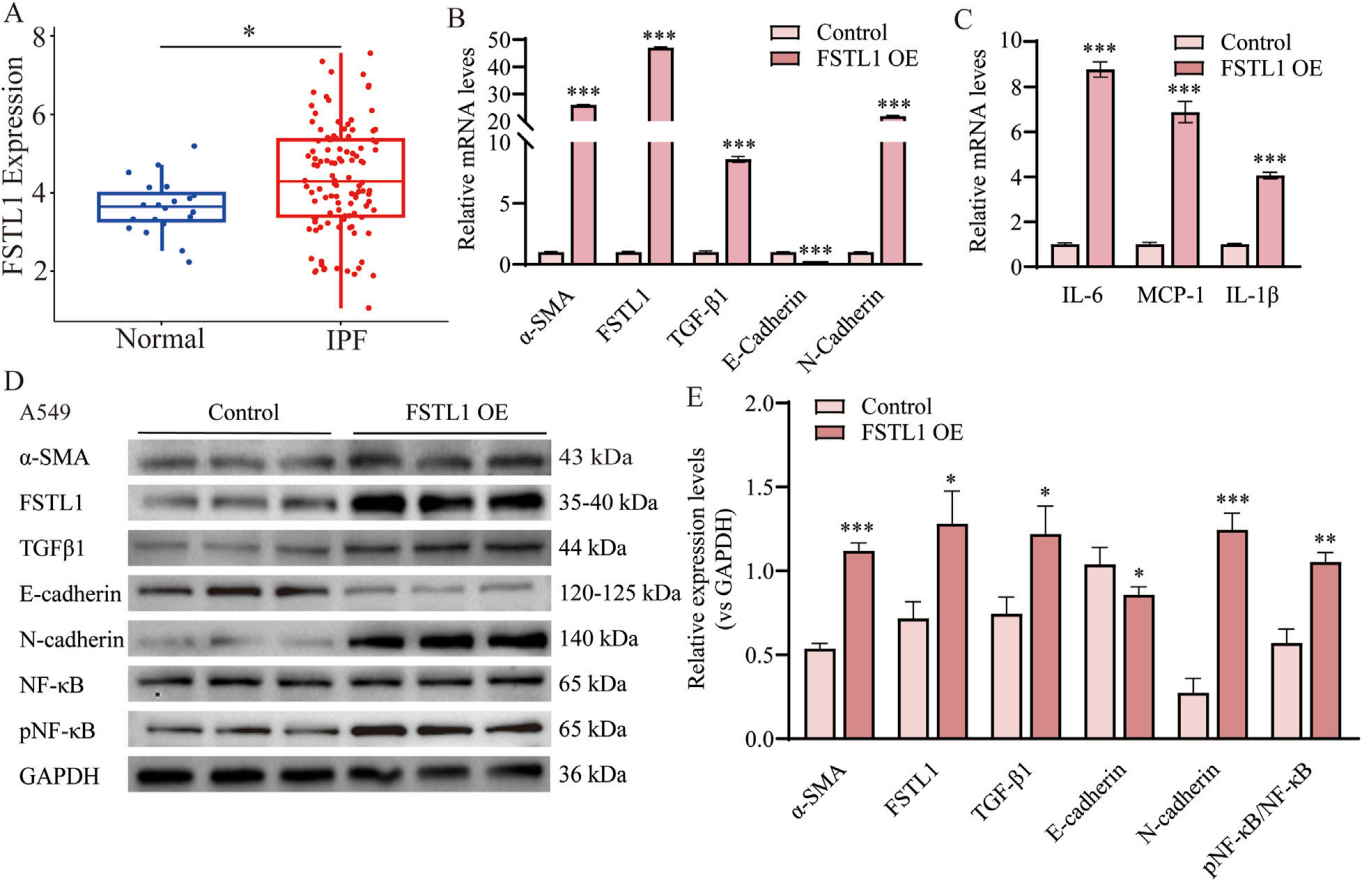
Overexpression of FSTL1 induces TGF-β1 expression and EMT in A549 cells. **(A)** Analysis of FSTL1 mRNA expression in bronchoalveolar lavage fluid from patients with idiopathic pulmonary fibrosis using the GSE70866 dataset from the GEO database. **(B)** Real-time qPCR analysis of mRNA expression, relative levels of α-SMA, FSTL1, TGF-β1, E-cadherin, and N-cadherin (n = 3). **(C)** Real-time qPCR analysis of mRNA expression, relative levels of IL-6, IL-1β, and MCP-1 (n = 3). **(D)** Protein expression levels of α-SMA, FSTL1, TGF-β1, E-cadherin, N-cadherin, total NF-κB, and phosphorylated NF-κB were determined using Western blotting. **(E)** Quantitative analysis of α-SMA, FSTL1, TGF-β1, E-cadherin, N-cadherin, total NF-κB, and phosphorylated NF-κB proteins was performed, with GAPDH as a loading control (n = 3). Data are presented as means ± SEM; *P < 0.05, **P < 0.01, and ***P < 0.001 compared with the control group. Abbreviations: IPF, idiopathic pulmonary fibrosis; FSTL1 OE, FSTL1 overexpression; TGF-β1, Transforming Growth Factor Beta 1; FSTL1, Follistatin-like 1; NF-κB, Nuclear Factor Kappa B; α-SMA, Alpha-Smooth Muscle Actin; IL-6, Interleukin-6; IL-1β, Interleukin-1β; pNF-κB, phosphorylated Nuclear Factor kappa-B; GAPDH, Glyceraldehyde-3-Phosphate Dehydrogenase; MCP-1, Monocyte Chemoattractant Protein-1.

Figure 4A illustrates data from the GEO database, which demonstrate a significant upregulation of FSTL1 expression in patients with IPF compared to normal controls, implying its potential involvement in the pathogenesis of this disease. To investigate the effect of FSTL1 on the NF-κB-mediated inflammatory pathway in the lungs, an FSTL1 overexpression model was established in A549 epithelial cells. The results revealed a clear feedback loop between FSTL1 and TGF-β1, with FSTL1 overexpression enhancing TGF-β1 expression levels (Figures 4B,D,E). Furthermore, FSTL1 overexpression significantly elevated the pNF-κB/NF-κB ratio (Figures 4D,E) and modulated the expression of inflammatory cytokines IL-6, IL-1β, and MCP-1 (Figure 4C). This regulatory relationship was further validated by bioinformatic analysis showing significant positive correlations between FSTL1 and core NF-κB pathway components (RELA, NFKB1) as well as downstream cytokines (IL-6, IL-1β, CCL2/MCP-1) (Supplementary Figure S2, Pearson correlation, *P < 0.05*). Additionally, FSTL1 overexpression promoted α-SMA and N-cadherin expression while reducing E-cadherin expression, suggesting that FSTL1 overexpression may also facilitate fibrosis in lung cells.

### 3.5 FSTL1 knockout attenuated TGF-β1-induced EMT and inflammation in A549 cells

**FIGURE 5:**
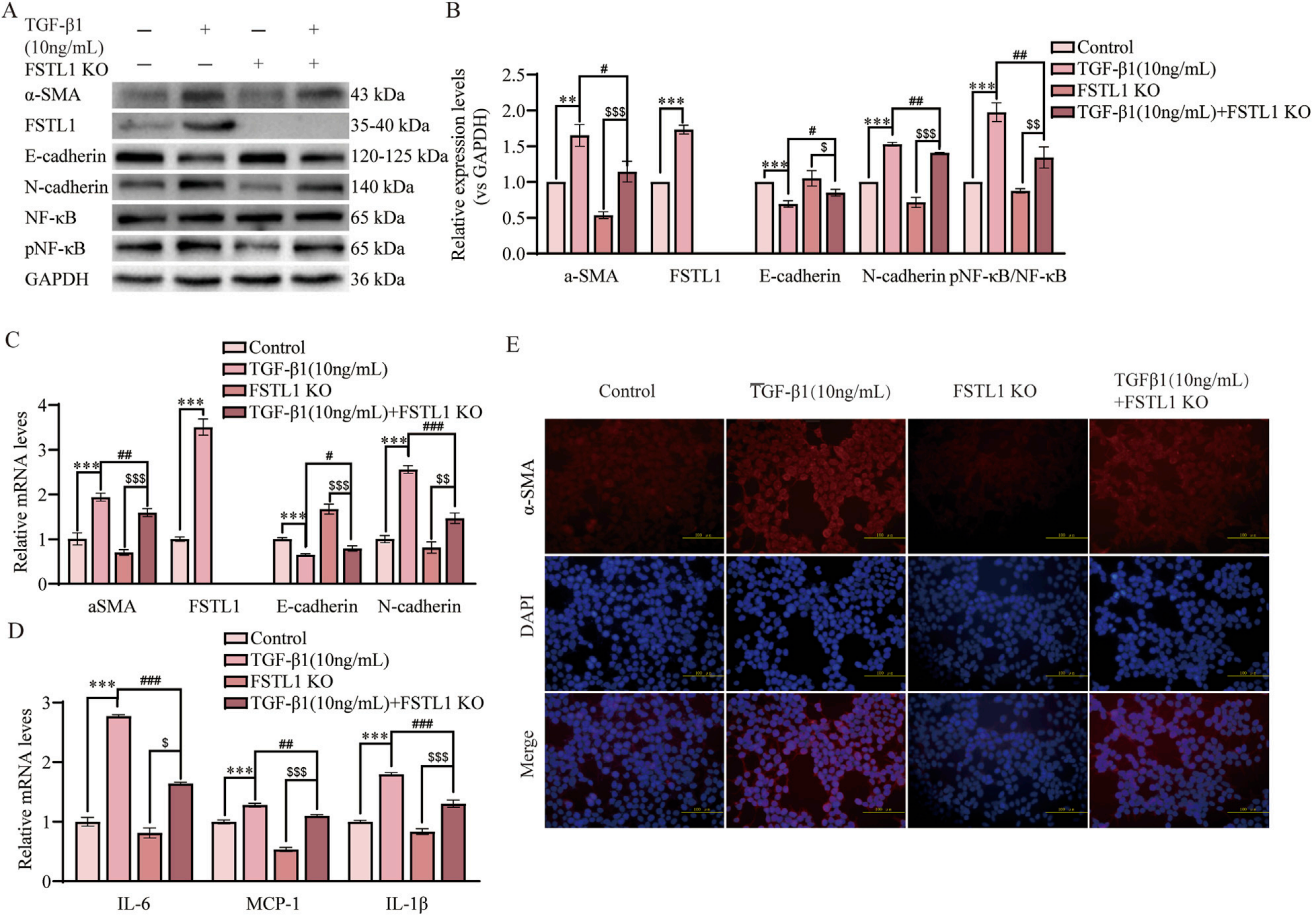
FSTL1 knockout attenuates TGF-β1-induced pulmonary fibrosis and inflammation in A549 cells. **(A)** Protein expression levels of α-SMA, FSTL1, E-cadherin, N-cadherin, total NF-κB, and phosphorylated NF-κB were determined using Western blotting. **(B)** Quantitative analysis of α-SMA, FSTL1, TGF-β1, E-cadherin, N-cadherin, total NF-κB, and phosphorylated NF-κB proteins was performed, with GAPDH as a loading control (n = 3). **(C)** Real-time qPCR analysis of mRNA expression, relative levels of α-SMA, FSTL1, E-cadherin, and N-cadherin (n = 3). **(D)** Real-time qPCR analysis of mRNA expression, relative levels of IL-6, IL-1β, and MCP-1 (n = 3). **(E)** Double immunofluorescence staining for FSTL1 and α-SMA in A549 cells, with a scale bar of 100 μm (n = 3). Data are presented as means ± SEM; *P < 0.05, **P < 0.01, and ***P < 0.001 compared with the control group; ^*#*^ *P < 0.05,* ^*##*^ *P < 0.01*, and ^*###*^ *P < 0.001* compared with the TGF-β1 group; ^*$*^ *P < 0.05,* ^*$$*^ *P < 0.01*, and ^*$$$*^ *P < 0.001* compared with the FSTL1 KO group. Abbreviations: FSTL1 KO, FSTL1 knockout; TGF-β1, Transforming Growth Factor Beta 1; FSTL1, Follistatin-like 1; NF-κB, Nuclear Factor Kappa B; α-SMA, Alpha-Smooth Muscle Actin; IL-6, Interleukin-6; IL-1β, Interleukin-1β; pNF-κB, phosphorylated Nuclear Factor kappa-B; GAPDH, Glyceraldehyde-3-Phosphate Dehydrogenase; MCP-1, Monocyte Chemoattractant Protein-1.

To evaluated the impact of FSTL1 gene knockout on TGF-β1-induced EMT and inflammatory responses. We utilized CRISPR/Cas9 to knockout FSTL1 in A549 cells, verifying the knockout via Western blot. Western blot analysis revealed that treatment with TGF-β1 significantly upregulated the expression of α-SMA and downregulated E-cadherin (Figures 5A,B). This effect was notably mitigated in the FSTL1 gene knockout group. Furthermore, the expression levels of FSTL1 and pNF-κB were increased, while N-cadherin was decreased in the TGF-β1 treatment group. These alterations were reversed upon FSTL1 gene knockout. qPCR analysis corroborated the changes at the mRNA level, demonstrating a significant upregulation of α-SMA, FSTL1, and N-cadherin mRNA in the TGF-β1 treatment group, with a corresponding decrease in E-cadherin mRNA (Figure 5C). Conversely, the FSTL1 gene knockout group exhibited an opposing trend. The mRNA levels of inflammatory cytokines IL-6, MCP-1, and IL-1β were significantly elevated in the TGF-β1 treatment group and were substantially reduced in the FSTL1 gene knockout group (Figure 5D). Immunofluorescence staining indicated that TGF-β1 treatment enhanced α-SMA expression, which was significantly diminished upon FSTL1 gene knockout (Figure 5E). Collectively, these findings demonstrate that FSTL1 gene knockout effectively attenuates TGF-β1-induced EMT and inflammatory responses, providing robust experimental evidence supporting FSTL1 as a potential therapeutic target.

### 3.6 Quercetin alleviated TGF-β1-induced EMT by suppressing FSTL1 expression and modulating the NF-κB signaling pathway *in vitro*

**FIGURE 6:**
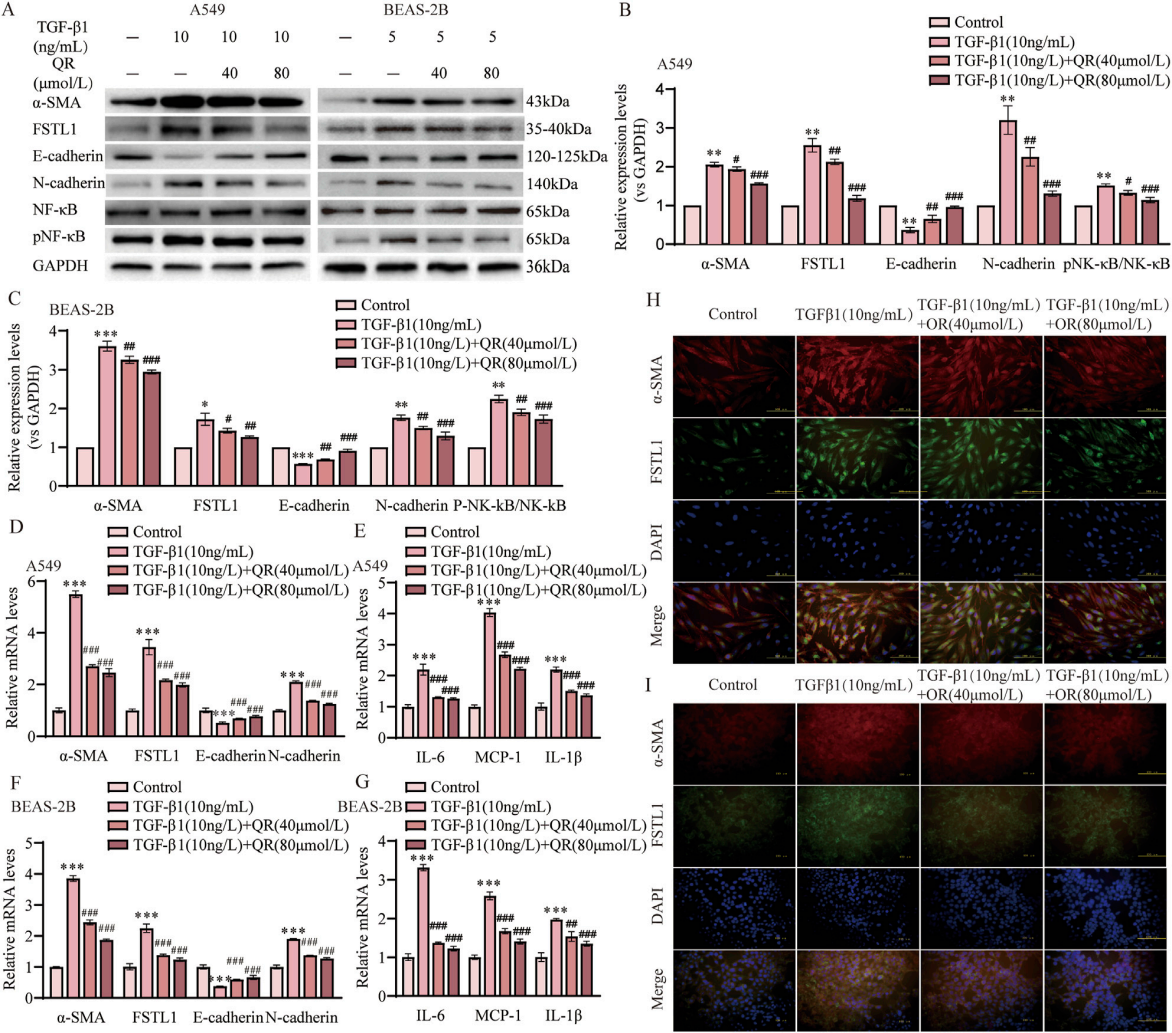
Quercetin alleviates TGF-β1-induced EMT via the FSTL1/NF-κB signaling pathway *in vitro*. **(A)** Protein expression levels of α-SMA, FSTL1, E-cadherin, N-cadherin, total NF-κB, and phosphorylated NF-κB were determined using Western blotting. **(B,C)** Quantitative analysis of α-SMA, FSTL1, E-cadherin, N-cadherin, total NF-κB, and phosphorylated NF-κB proteins in A549 and BEAS-2B cells, with GAPDH as a loading control (n = 3). **(D)** Real-time qPCR analysis of mRNA expression, relative levels of α-SMA, FSTL1, E-cadherin, and N-cadherin in A549 cells (n = 3). **(E)** Real-time qPCR analysis of mRNA expression, relative levels of IL-6, IL-1β, and MCP-1 in A549 cells (n = 3). **(F)** Real-time qPCR analysis of mRNA expression, relative levels of α-SMA, FSTL1, E-cadherin, and N-cadherin in BEAS-2B cells (n = 3). **(G)** Real-time qPCR analysis of mRNA expression, relative levels of IL-6, IL-1β, and MCP-1 in BEAS-2B cells (n = 3). **(H)** Double immunofluorescence staining for FSTL1 and α-SMA in A549 cells, with a scale bar of 100 μm (n = 3). **(I)** Double immunofluorescence staining for FSTL1 and α-SMA in BEAS-2B cells, with a scale bar of 100 μm (n = 3). Data are presented as means ± SEM; *P < 0.05, **P < 0.01, and ***P < 0.001 compared with the control group; ^*#*^ *P < 0.05,* ^*##*^ *P < 0.01,* and ^*###*^ *P < 0.001* compared with the TGF-β1 group. Abbreviations: TGF-β1, Transforming Growth Factor Beta 1; FSTL1, Follistatin-like 1; NF-κB, Nuclear Factor Kappa B; α-SMA, Alpha-Smooth Muscle Actin; IL-6, Interleukin-6; IL-1β, Interleukin-1β; MCP-1, Monocyte Chemoattractant Protein1; pNF-κB, phosphorylated Nuclear Factor kappa-B; GAPDH, Glyceraldehyde-3-Phosphate Dehydrogenase; QR, Quercetin.

To determine the optimal timing and concentration of quercetin for mitigating TGF-β1-induced EMT and FSTL1 pathway activation *in vitro*, we employed a comparative analysis in A549 and BEAS-2B cell lines. Two distinct treatment regimens were evaluated: concurrent administration of quercetin and TGF-β1, and quercetin administration 24 h post TGF-β1 to intervene after EMT initiation. A dose-response curve was established with quercetin concentrations from 0 to 100 μmol/L. Our findings demonstrate that TGF-β1 significantly upregulated α-SMA and downregulated E-cadherin expression, indicative of EMT. Quercetin’s influence was dose-dependent, with a notable reduction in α-SMA and restoration of E-cadherin at 40 μmol/L in both cell lines (Supplementary Figure S1A,D). In the second administration method, the inhibitory effect of quercetin on α-SMA and FSTL1 expression plateaued beyond 40 μmol/L, suggesting a threshold effect in A549 cells (Supplementary Figure S1B,C). The inhibitory effects of quercetin on the expression of α-SMA, E-cadherin, and FSTL1 in BEAS-2B cells tend to stabilize at concentrations above 80 μmol/L (Supplementary Figure S1D–F). Consequently, quercetin concentrations of 40 and 80 μmol/L from the second treatment protocol were chosen for subsequent studies. Post-treatment with quercetin, a significant downregulation of FSTL1 was observed compared to TGF-β1 alone (Figures 6A–D,F). Moreover, quercetin inhibited the release of inflammatory factors, including IL-6, IL-1β, and MCP-1, during the EMT process (Figures 6E,G). Cell fluorescence experiments confirmed these findings: after TGF-β1 treatment, strong fluorescence signals for α-SMA and FSTL1 were observed in the cytoplasm, but these signals were significantly diminished following quercetin treatment, consistent with qPCR and Western blot results (Figures 6H,I). In conclusion, the regulatory effects of quercetin on EMT and inflammation may be linked to its ability to reduce FSTL1 expression via the NF-κB signaling pathway *in vitro*.

### 3.7 FSTL1 mediates quercetin’s anti-fibrotic effects in TGF-β1-treated A549 cells

**FIGURE 7:**
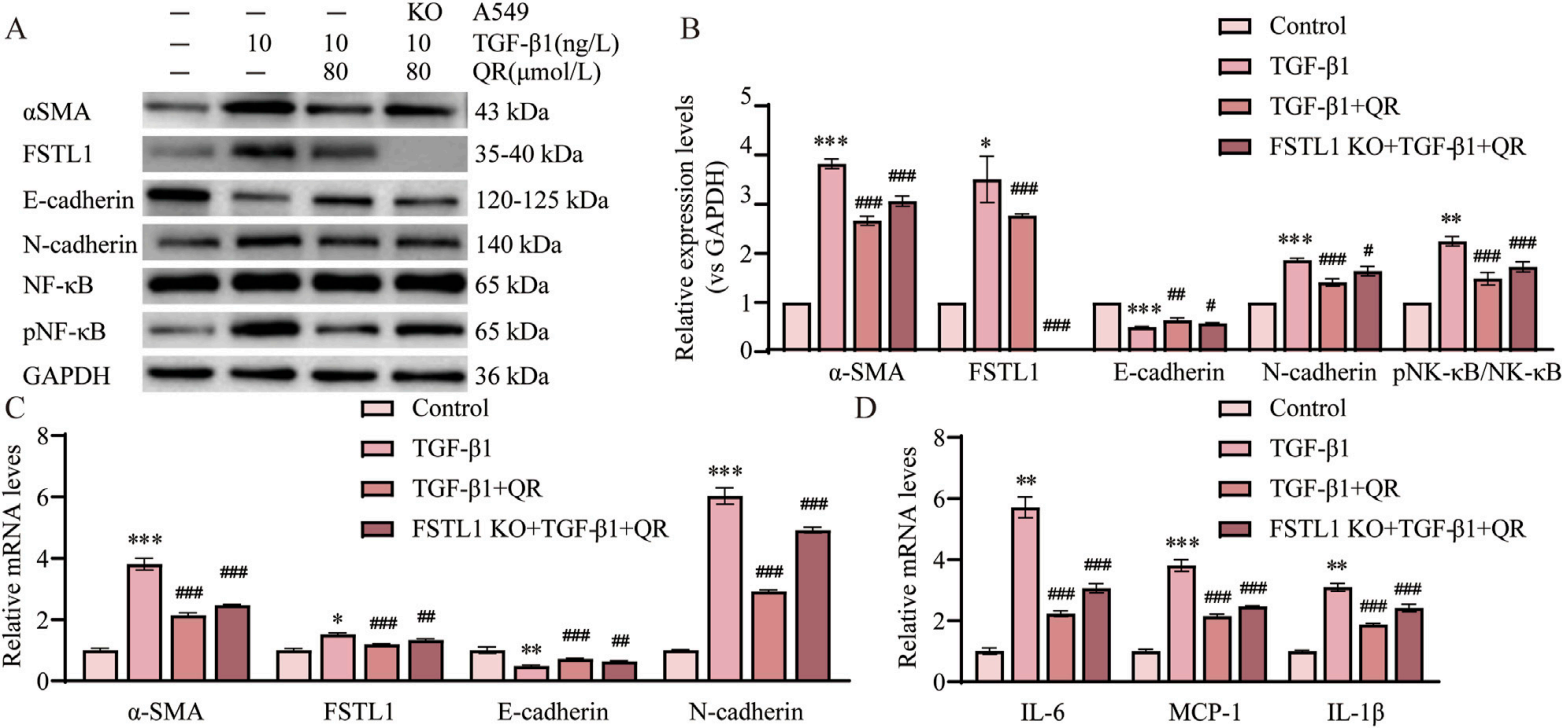
FSTL1 knockout abrogates the anti-fibrotic and anti-inflammatory effects of quercetin in A549 cells. **(A)** Protein expression levels of α-SMA, FSTL1, E-cadherin, N-cadherin, total NF-κB, and phosphorylated NF-κB were determined using Western blotting. **(B)** Quantitative analysis of α-SMA, FSTL1, E-cadherin, N-cadherin, total NF-κB, and phosphorylated NF-κB proteins in A549 cells, with GAPDH as a loading control (n = 3). **(C)** Real-time qPCR analysis of mRNA expression, relative levels of α-SMA, FSTL1, E-cadherin, and N-cadherin (n = 3). **(D)** Real-time qPCR analysis of mRNA expression, relative levels of IL-6, IL-1β, and MCP-1 (n = 3). Data are presented as means ± SEM; *P < 0.05, **P < 0.01, and ***P < 0.001 compared with the control group; ^*#*^ *P < 0.05,* ^*##*^ *P < 0.01*, and ^*###*^ *P < 0.001* compared with the TGF-β1 group. Abbreviations: TGF-β1, Transforming Growth Factor Beta 1; FSTL1, Follistatin-like 1; NF-κB, Nuclear Factor Kappa B; α-SMA, Alpha-Smooth Muscle Actin; IL-6, Interleukin 6; IL-1β, Interleukin 1 Beta; MCP-1, Monocyte Chemoattractant Protein 1; pNF-κB, phosphorylated Nuclear Factor kappa-B; GAPDH, Glyceraldehyde-3-Phosphate Dehydrogenase; QR, Quercetin.

Results showed that FSTL1 knockout markedly reduced quercetin’s inhibition of the p-NF-κB/NF-κB ratio and lessened the downregulation of IL-1β, IL-6, and MCP-1 (Figures 7A,B,D). These findings confirm quercetin’s anti-inflammatory action in pulmonary fibrosis through TGF-β1-induced NF-κB pathway inhibition, which FSTL1 knockout partially negates. Furthermore, FSTL1 knockout partially reversed quercetin’s impact on α-SMA, E-cadherin, and N-cadherin levels (Figures 7A–C). Critical rescue experiments confirmed that quercetin treatment failed to attenuate fibrotic responses in FSTL1-overexpressing (FSTL1-OE) groups, where TGF-β1-treated A549 cells exhibited sustained elevation of p-NF-κB/NF-κB activation, upregulated inflammatory cytokines (IL-1β, IL-6, MCP-1), and increased EMT marker levels (α-SMA, N-cadherin, E-cadherin)—with quercetin administration providing no significant reduction in NF-κB pathway activity, inflammatory mediator expression, or EMT dysregulation compared to FSTL1-OE controls (Supplementary Figure S3A–D). Collectively, these findings highlight FSTL1’s pivotal role in mediating quercetin’s anti-fibrotic effects, offering new perspectives on quercetin’s therapeutic potential in pulmonary fibrosis.

## 4 Discussion

Pulmonary fibrosis is a chronic, progressive interstitial lung disease characterized by the replacement of normal lung tissue with abnormal fibrous tissue, leading to a gradual decline in lung function. Quercetin mitigates pulmonary fibrosis by suppressing EMT and inflammation, yet its regulation of FSTL1/NF-κB axis remains unexplored. This study demonstrates that quercetin disrupts the FSTL1-TGF-β1 feedback loop, inhibiting NF-κB-driven epithelial-to-mesenchymal transition (EMT) and cytokine release. These findings provide novel insights into the mechanisms underlying quercetin’s potential therapeutic role in pulmonary fibrosis.

Previous studies demonstrate quercetin’s efficacy in improving alveolar architecture and reducing collagen deposition in pulmonary fibrosis animal models (Boots et al., 2020; Reyes-Jimenez et al., 2023), consistent with our findings. Mechanistically, quercetin counteracts fibrosis through TGF-β1-SMAD2/3 inhibition (suppressing myofibroblast differentiation), Nrf2 activation (enhancing antioxidant defenses), and attenuation of macrophage senescence/SASP-mediated inflammation (Geng et al., 2022; Geng et al., 2023). These preclinical effects translate clinically, where 1250 mg/day quercetin potentiates dasatinib’s senolytic action to clear senescent cells and improve patient mobility in pulmonary fibrosis trials (Nambiar et al., 2023). Pulmonary fibrosis is characterized by excessive ECM accumulation, with EMT playing a crucial role in organ fibrosis, particularly in the context of chronic inflammation (Saadh et al., 2024). This process is characterized by increased α-SMA and N-cadherin expression, along with a loss of E-cadherin, indicative of excessive epithelial-to-mesenchymal transition. Quercetin suppresses EMT-driven malignancy across carcinomas through tissue-specific mechanisms: in breast cancer by modulating the circHIAT1/miR-19a-3p/CADM2 axis, in lung cancer via Akt/MAPK pathway inhibition that reduces β-catenin nuclear translocation, and in oral squamous cell carcinoma through blockade of TGF-β1-induced EMT, collectively inhibiting metastatic phenotypes (Li et al., 2024; Elumalai et al., 2022; Kim et al., 2020). Notably, in our TGF-β1-stimulated A549/BEAS-2B *in vitro* EMT model, quercetin attenuated pulmonary fibrosis progression by effectively inhibiting EMT. This aligns with established crosstalk between fibrosis and EMT pathways, where cytokines (e.g., TNF-α/NF-κB and IL-6/STAT3 axes) and microbial factors coregulate EMT through shared signaling nodes like TGF-β (Wree et al., 2018; Pedroza et al., 2016; Jakubzick et al., 2003; Liu, 2008). Crucially, quercetin specifically suppressed EMT-associated NF-κB activation and reduced IL-1β/IL-6/IL-8 release, demonstrating targeted anti-inflammatory efficacy within the fibrotic microenvironment. While our study focused on epithelial cells (A549/BEAS-2B) as the primary research model—driven by the central role of alveolar epithelial injury and EMT initiation in IPF pathogenesis—the observed *in vivo* suppression of macrophage-derived cytokines (IL-1β, MCP-1, IL-6) and collagen deposition suggests broader mechanistic implications. Specifically, quercetin’s inhibition of NF-κB signaling within epithelial cells likely disrupts crosstalk with immune cells, dampening macrophage-mediated inflammation. Furthermore, by blocking EMT (a key source of activated fibroblasts) and reducing profibrotic cytokine release (e.g., TGF-β1, IL-6), quercetin indirectly attenuates fibroblast activation and ECM deposition, as evidenced by our BLM model data.

Follistatin-like protein 1 (FSTL1) is a secreted glycoprotein highly homologous to follistatin, belonging to the SPARC (secreted protein acidic and rich in cysteine) family of cysteine-rich, acidic secretory proteins. It contains a follistatin-like domain and a calcium-binding domain. FSTL1 is involved in various fibrotic processes. For example, in liver diseases, FSTL1 exacerbates fibrosis by promoting inflammatory macrophage polarization and attenuating mesenchymal stem cell (MSC)-mediated immunosuppression (Sun et al., 2023; Gu et al., 2023; Zheng et al., 2022). Notably, FSTL1 has been shown to induce EMT in skin squamous cell carcinoma by interacting with Zinc finger E-box binding homeobox 1 (ZEB1), enhancing tumor cell proliferation, migration, and invasion (Yu et al., 2024). However, the role of FSTL1 in regulating EMT in pulmonary fibrosis remains unclear. Here, we reveal a novel FSTL1-TGF-β1 positive feedback loop in lung epithelial cells: TGF-β1 induces FSTL1, which further amplifies TGF-β1 and NF-κB activation. Critically, FSTL1 knockout suppressed EMT markers (α-SMA and N-cadherin), establishing FSTL1 as a pivotal EMT driver in pulmonary fibrosis. FSTL1’s pro-inflammatory role—mediated through NF-κB activation—is established across diseases. In osteonecrosis, elevated FSTL1 promotes NF-κB-dependent cytokine release and tissue degradation (Qu et al., 2019), while in infections and chondrocytes, it drives inflammation via TLR4/NF-κB and MMP induction (Hu et al., 2019; Chen and Liu, 2019). Furthermore, downregulation of FSTL1 reduces the expression of inflammatory cytokines such as IL-1β, TNF-α, and IL-6 (Liu et al., 2017). Critically, our study extends this paradigm to pulmonary fibrosis: FSTL1 overexpression in A549 cells amplified NF-κB and cytokines (IL-1β/TNF-α/IL-6), FSTL1 inhibition attenuated TGF-β1-induced inflammation. Given quercetin’s known anti-inflammatory and MMP-inhibitory effects (Sun et al., 2021; Wang et al., 2023), we propose it targets FSTL1. This work provides the first evidence that quercetin suppresses FSTL1 expression, elucidating a novel antifibrotic mechanism.

Although our study demonstrates that quercetin alleviates pulmonary fibrosis through FSTL1-mediated suppression of NF-κB signaling, three key limitations warrant consideration. First, potential crosstalk with other profibrotic pathways—notably Wnt/β-catenin and JAK-STAT—remains unvalidated, leaving open the possibility of parallel or compensatory mechanisms. Second, the absence of direct comparison with clinical standard-of-care agents (e.g., pirfenidone or nintedanib) precludes definitive conclusions about quercetin’s relative efficacy, as our data only establish its therapeutic potential against pathological controls rather than benchmark therapies. Third, while our epithelial-focused approach directly addresses the FSTL1/NF-κB/EMT axis-a cornerstone of IPF initiation—the lack of direct experimentation on macrophages or fibroblasts precludes definitive conclusions about cell-type-specific effects. Additionally, quercetin’s inherent pharmacokinetic limitations (low oral bioavailability and rapid metabolism) may underestimate its true antifibrotic potential in our model. Future studies should prioritize: (1) mechanistic dissection of FSTL1’s interaction with alternative pathways; (2) head-to-head efficacy trials against approved antifibrotics; and (3) development of targeted delivery systems (e.g., nanoparticle-encapsulated quercetin) to overcome bioavailability constraints.

In conclusion, this study demonstrates that quercetin, a natural compound, significantly improves the inflammatory response and EMT process in BLM-induced pulmonary fibrosis by suppressing FSTL1 expression and modulating the NF-κB signaling pathway. These findings offer novel insights into the mechanisms through which quercetin alleviates pulmonary fibrosis, presenting it as a potential therapeutic candidate for this disease.

## 5 Conclusion

In conclusion, Quercetin demonstrated therapeutic potential in treating pulmonary fibrosis by mitigating lung damage and inflammation *in vivo*. It suppressed EMT via downregulating FSTL1 and modulating the NF-κB pathway. *In vitro* studies confirmed FSTL1’s role in TGF-β1-induced EMT, with Quercetin reversing these effects. These findings suggest Quercetin’s mechanism of action and its potential as a treatment for pulmonary fibrosis.

## Data Availability

The original contributions presented in the study are included in the article/Supplementary Material, further inquiries can be directed to the corresponding authors.

## Supplementary material

SUPPLEMENTARY FIGURE 1 Quercetin dosing and timing in TGF-β1-induced pulmonary fibrosis and FSTL1 expression. **(A-C)** Real-time qPCR analysis of mRNA expression, relative levels of α-SMA, FSTL1, and E-cadherin with TGF-β1 and Quercetin, under simultaneous and delayed Quercetin treatment regimens in A549 cells. (n = 3). **(D-F)** Real-time qPCR analysis of mRNA expression, relative levels of α-SMA, FSTL1, and E-cadherin with TGF-β1 and Quercetin, under simultaneous and delayed Quercetin treatment regimens in BEAS-2B cells. (n = 3). Data are presented as means ± SEM; *P < 0.05, **P < 0.01, and ***P < 0.001 compared with the A549 cells control group; #P < 0.05, ##P < 0.01, and ###P < 0.001 compared with the BEAS-2B cells control group. Abbreviations: TGF-β1, Transforming Growth Factor Beta 1; QR, Quercetin; α-SMA, Alpha-Smooth Muscle Actin; FSTL1, Follistatin-like 1.

SUPPLEMENTARY FIGURE 2 Co-expression Heatmap of FSTL1-Associated Genes. **(A)** Hierarchical clustering heatmap displaying the Pearson correlation coefficient matrix between FSTL1 and NF-κB pathway genes. Color gradient ranges from blue (r = 0) to red (r = 1), with white indicating intermediate correlation (r = 0.5). Asterisks within cells denote statistical significance levels (**p < 0.05, **p < 0.01*, ****p < 0.001*).

SUPPLEMENTARY FIGURE 3 FSTL1 overexpression rescues quercetin’s suppression of TGF-β1-induced EMT and inflammation via the FSTL1/NF-κB pathway in A549 cells. **(A)** Protein expression levels of α-SMA, FSTL1, E-cadherin, N-cadherin were assessed by Western blotting. **(B)** Quantitative analysis of α-SMA, FSTL1, E-cadherin, N-cadherin proteins was performed, with GAPDH used as a loading control (n = 3). **(C)** Real-time qPCR analysis of mRNA expression, relative levels of α-SMA, FSTL1, E-cadherin, and N-cadherin (n = 3). **(D)** Real-time qPCR analysis of mRNA expression, relative levels of IL-6, IL-1β, and MCP-1 (n = 3). Data are presented as means ± SEM; **P < 0.05, **P < 0.01*, ****P < 0.001* compared with the control group; ^*#*^ *P < 0.05,* ^*##*^ *P < 0.01,* ^*###*^ *P < 0.001* compared with the TGF-β1+QR group; ^*$*^ *P < 0.05,* ^*$$*^ *P < 0.01*, and ^*$$$*^ *P < 0.001* compared with the FSTL1 OE TGF-β1 group. Abbreviations: TGF-β1, Transforming Growth Factor Beta 1; OE, overexpression; NF-κB, Nuclear Factor Kappa B; p-NF-κB, phosphorylated NF-κB; α-SMA, Alpha-Smooth Muscle Actin; EMT, epithelial-mesenchymal transition; IL, interleukin; MCP-1, Monocyte Chemoattractant Protein 1; QR, Quercetin.
