# Supplementary figures and images for: Quercetin ameliorates epithelial-mesenchymal transition and inflammation by targeting FSTL1 and modulating the NF-κB pathway in pulmonary fibrosis
Source: Front Pharmacol. 2025 Jul 30;16:1594757. doi: 10.3389/fphar.2025.1594757 (PMC12343526; doi:10.3389/fphar.2025.1594757)

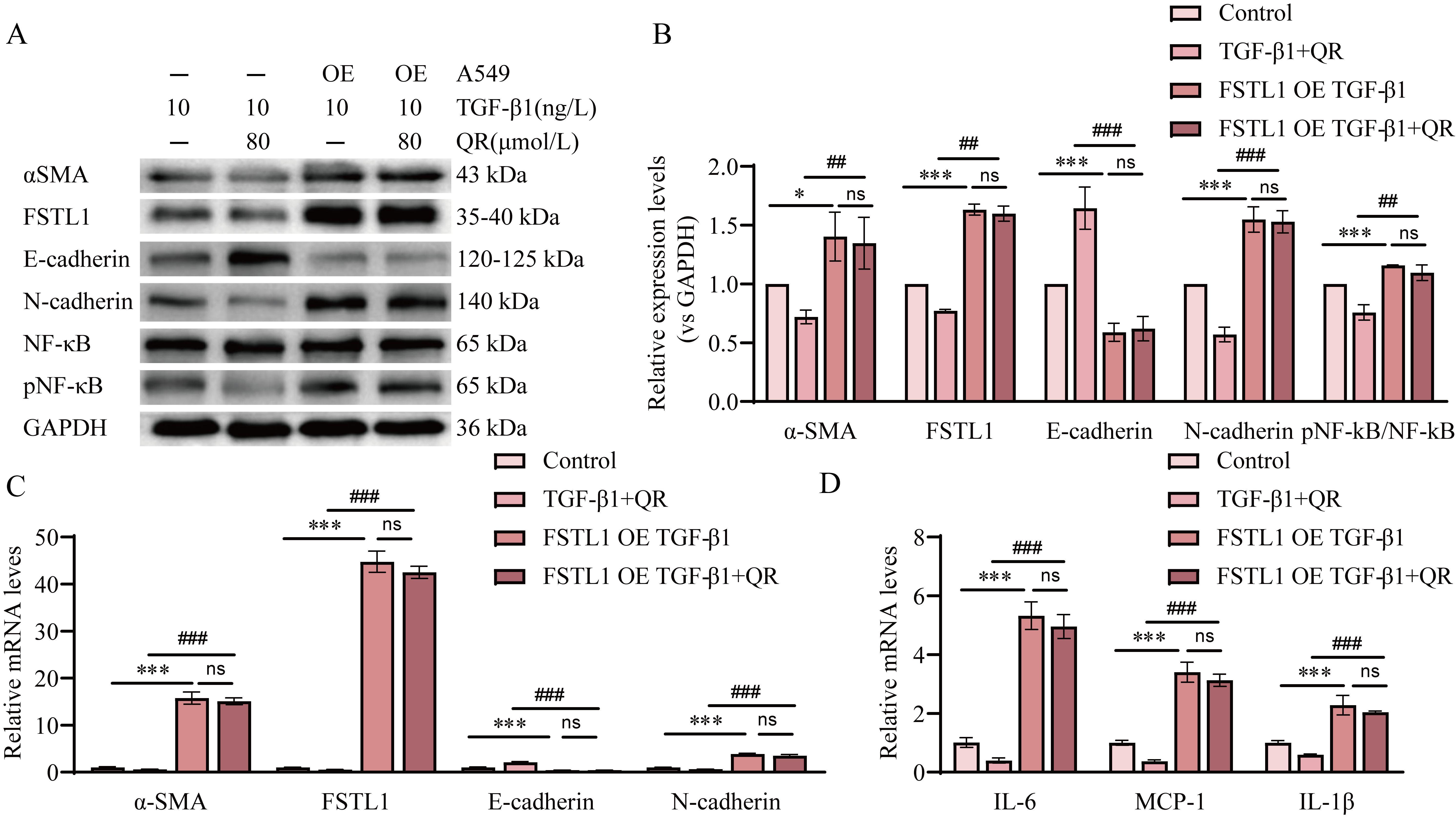

Supplement: Supplementary file 1 [file Image3.tif]

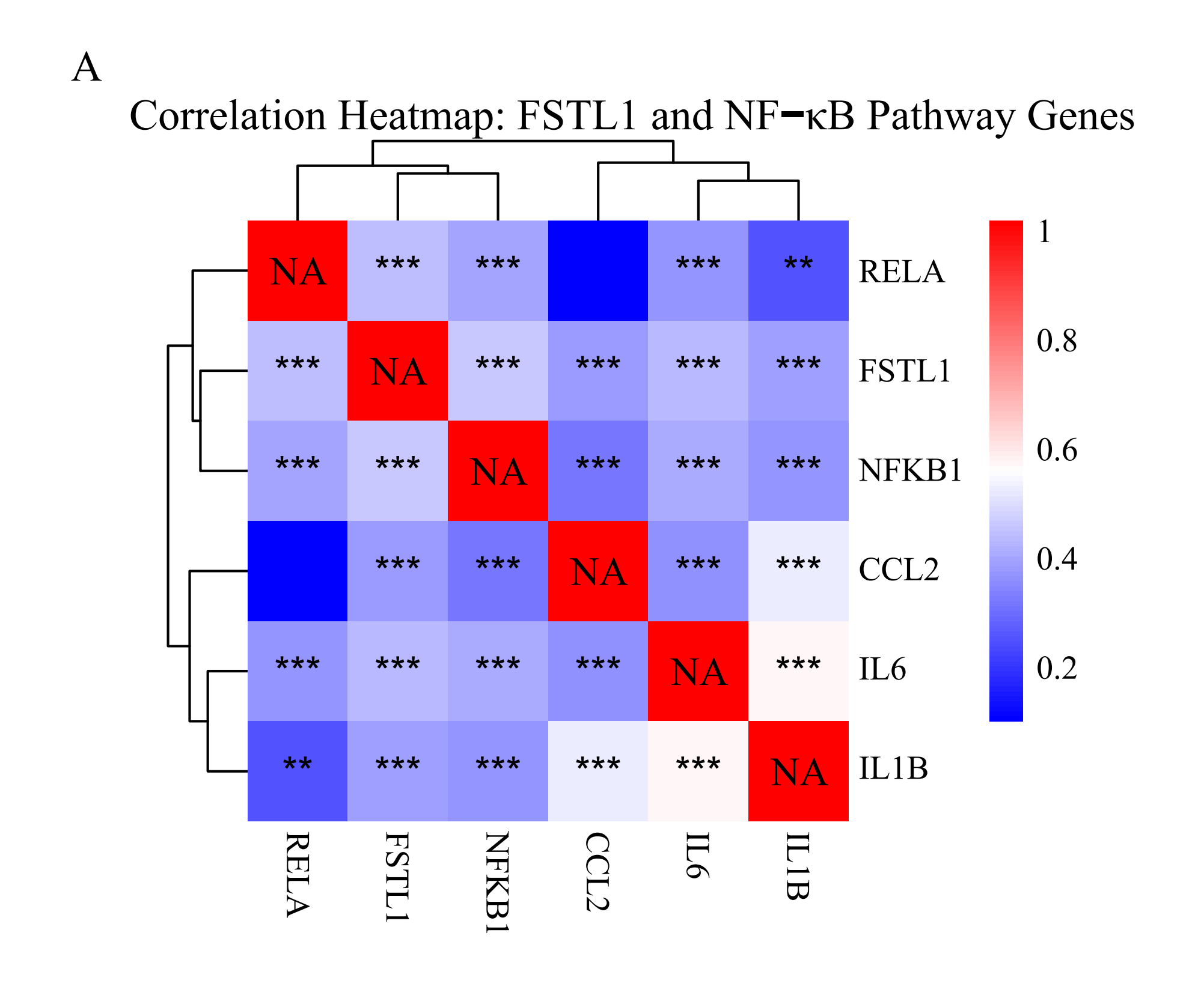

Supplement: Supplementary file 2 [file Image2.tif]

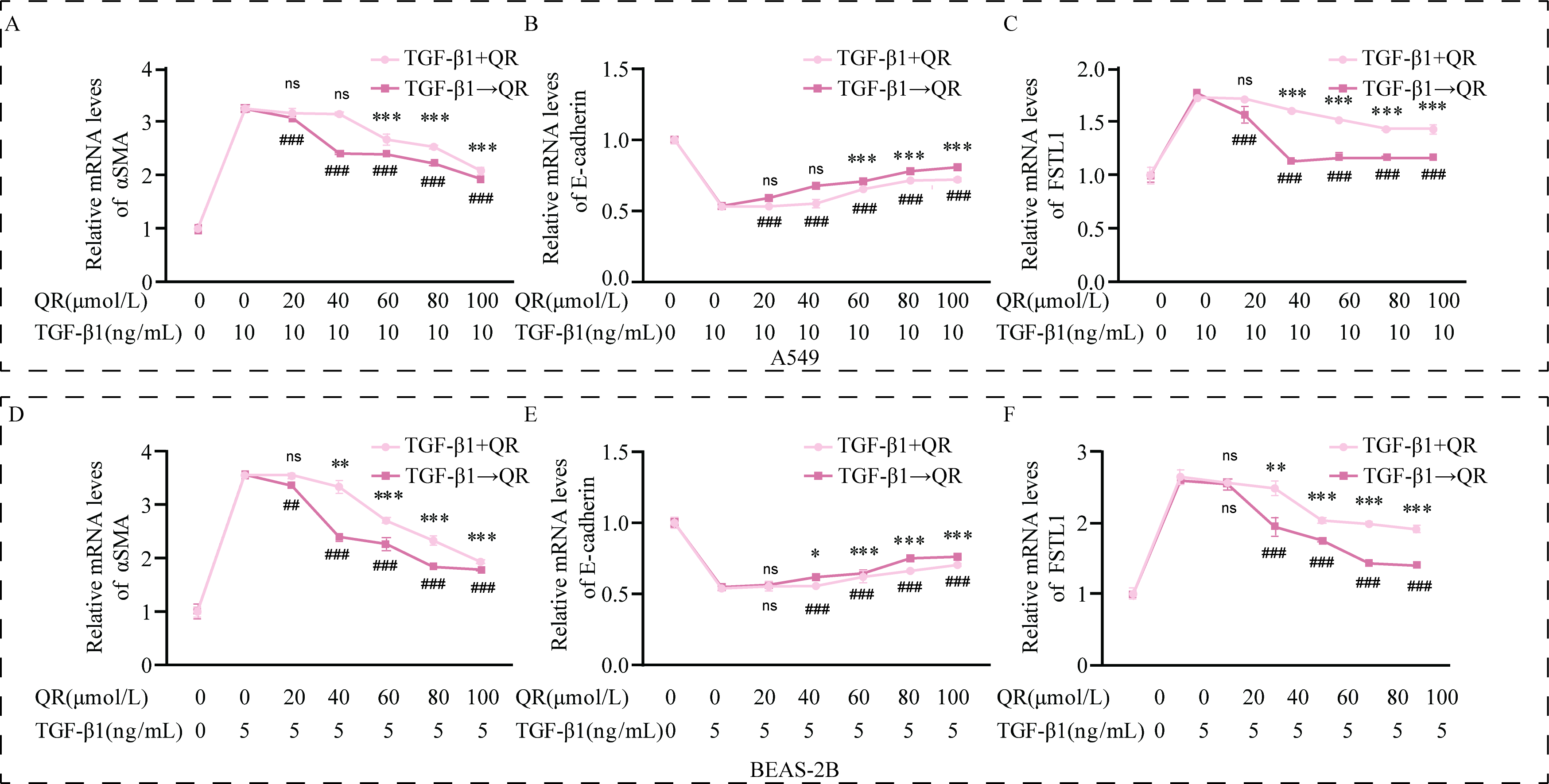

Supplement: Supplementary file 3 [file Image1.tif]
